# Transcriptomic and Immunopathological Profiles of Inflammasomes in Different Clinical Forms of American Cutaneous Leishmaniasis

**DOI:** 10.3390/microorganisms13050980

**Published:** 2025-04-24

**Authors:** Larissa dos Santos Alcântara, Marliane Batista Campos, Ana Carolina Stocco Lima, Alessandra Pontillo, Kamilla Batista da Silva Souza, Aurea Favero Ferreira, Cristina Pires Camargo, Sueli Mieko Oba-Shinjo, Márcia Dalastra Laurenti, Carlos Eduardo Pereira Corbett, Vania L. R. da Matta, Helder Nakaya, Fernando T. Silveira, Claudia Maria de Castro Gomes

**Affiliations:** 1 Laboratorio de Patologia de Moléstias Infecciosas—LIM-50 Departamento de Patologia, Hospital das Clinicas HCFMUSP, Faculdade de Medicina, Universidade de São Paulo, Sao Paolo 01246-903, SP, Brazil; aurea.favero@gmail.com (A.F.F.); mdlauren@usp.br (M.D.L.); ccorbett@usp.br (C.E.P.C.); mattav@usp.br (V.L.R.d.M.); 2 Instituto Evandro Chagas, Departamento de Parasitologia, Belém 70300-000, PA, Brazil; marlianecampos@iec.gov.br (M.B.C.); carolstocco@gmail.com (A.C.S.L.); fernandotobias@iec.gov.br (F.T.S.); 3 Laboratório de Imunogenética, Departamento de Imunologia, Instituto de Ciências Biomédicas, Universidade de São Paulo, Sao Paolo 05508-000, SP, Brazil; pontillo.a@gmail.com (A.P.); kamillasouza@usp.br (K.B.d.S.S.); 4 Laboratorio de Microcirurgia—Cirurgia Plástica—LIM-04 Hospital das Clinicas HCFMUSP, Faculdade de Medicina, Universidade de São Paulo, Sao Paolo 01246-903, SP, Brazil; cristinacamargo@usp.br; 5 Laboratório de Biologia Celular e Molecular—LIM-15 Departamento de Neurologia, Hospital das Clinicas HCFMUSP, Faculdade de Medicina, Universidade de São Paulo, Sao Paolo 01246-903, SP, Brazil; suelimoba@usp.br; 6 Departamento de Análises Clínicas e Toxicológicas, Faculdade de Ciencias Farmaceuticas, Universidade de São Paulo, Sao Paolo 05508-220, SP, Brazil; hnakaya@usp.br; 7 Hospital Israelita Albert Einstein, Sao Paolo 05652-900, SP, Brazil; 8 Núcleo de Medicina Tropical, Universidade Federal do Pará, Belém 67030-000, PA, Brazil

**Keywords:** american cutaneous leishmaniasis, inflammasomes, transcriptomic, immunopathological profiles, *Leishmania (Leishmania) amazonensis*, *Leishmania (Viannia) braziliensis*

## Abstract

American cutaneous leishmaniasis (ACL), caused by *Leishmania (Leishmania) amazonensis* and *L. (Viannia) braziliensis*, presents a wide spectrum of clinical and immunopathological manifestations, ranging from localized cutaneous leishmaniasis (LCL) to severe forms like anergic diffuse cutaneous (ADCL) and mucocutaneous leishmaniasis (MCL). Despite evidence of the immune response’s complexity, the role of inflammasomes in disease severity and parasite persistence remains unclear. We investigated the transcriptomic and immunopathological profiles of inflammasome components in patient lesions across the clinical spectrum. Genes such as *NLRP3*, *AIM2*, *NLRP12*, *NLRC4*, *CASP1*, *CASP5*, *GSDMD*, and *IL1B* and all evaluated proteins, showed higher expression in ACL compared to healthy controls. Distinct inflammasome activation patterns were observed: MCL, the hyperreactive form, showed elevated *NLRP3*, *AIM2*, and *IL-1β*, indicating an intensified inflammatory environment. ADCL, the hyporeactive form, displayed increased *NLRP12* and *NLRC4* expression with reduced *GSDMD*. Localized forms showed transitional profiles, highlighting ACL’s multifactorial pathogenesis. These findings advance our understanding of inflammasome mechanisms in ACL, identifying potential therapeutic targets to modulate inflammation and improve management.

## 1. Introduction

Leishmaniasis is an infectious parasitic disease caused by more than twenty species of protozoa of the genus Leishmania, belonging to the subfamily Leishmaniinae [1]. These parasites are distributed among the three subgenera: *L. (Leishmania)*, *L. (Viannia)*, and *L. (Mundinia)* [2,3]. In Brazil, seven species are known to cause American cutaneous leishmaniasis (ACL), with *Leishmania (L.) amazonensis* and *Leishmania (V.) braziliensis* being of greatest clinical and epidemiological interest due to their increased pathogenic potential in humans [3,4].

ACL can present a range of clinical forms, comprising a spectrum that extends from localized cutaneous leishmaniasis (LCL)—caused by the *L. (Leishmania)* subgenus and the *L. (Viannia)* subgenus—to the polar and more severe forms of the disease: anergic diffuse cutaneous leishmaniasis (ADCL), caused by the *L. (Leishmania)* subgenus, and mucocutaneous or mucosal leishmaniasis (MCL/ML), caused by the *L. (Viannia)* subgenus. Patients with LCL exhibit a balanced Th1/Th2 cellular immune response and moderate positivity by the delayed type-hypersensitivity test (DTH) [3,4,5]. Conversely, if there is an imbalance in the cellular immune response, the patient may develop one of the polar forms of the disease. The hyporeactive pole (ADCL) is characterized by absent or weak DTH reactivity, predominantly a Th2-type cellular immune response, and high production of IL-4, IL-10, and TGF-β. In the hyperreactive pole (MCL), patients exhibit strong DTH reactivity, an exacerbated Th1-type cellular immune response, and increased production of IFN-γ and TNF-α, resulting in lesions in the nasal septum or palate [3].

Following infection by *Leishmania* spp., inflammasomes play a crucial role in the development of the host’s cellular immune response [6,7]. An inflammasome is a cytosolic multiprotein complex that senses pathogens or cell stress and responds with the activation of caspase-1 (canonical activation) or caspase-5 (non-canonical activation) and the consequent release of the mature forms of pro-inflammatory cytokines IL-1β and IL-18, and of gasdermin-D (GSDMD) [6]. The IL-1β derived from NLRP3 inflammasome activation is associated with host resistance in experimental *Leishmania* spp. infections through nitric oxide production [8]. On the other hand, in mice infected with *L. (L.) major*, this cytokine has been associated with host susceptibility [6]. In contrast, IL-18 has been shown to promote a Th2 response and host susceptibility in experimental infections with *L. (L.) mexicana* and *L. (L.) amazonensis* [9,10].

The cleaved form of GSDMD (N-GSDMD) produces pores in cellular membranes, allowing the release of IL-1β and IL-18 or, depending on the cell type and the intensity of the stimulus, a programmed inflammatory cell death known as pyroptosis. Pyroptosis has been proposed to be a response against intracellular pathogens [11]. In the context of leishmaniasis, Sá et al. [12] investigated how *Leishmania* protozoa manipulate GSDMD activation to favor persistent infection. The parasite interferes with GSDMD activation, inhibiting pyroptosis and allowing its survival within macrophages, thus contributing to the persistence of the infection in the host.

Several cytosolic innate receptors can activate the complex, such as NLRP3, AIM2, NLRP12, and NLRC4, which have been studied in murine models of *Leishmania* infection. In mice the NLRP3 inflammasome can either contribute to persistent infection or disease control, depending on the parasite species [7,13,14]. Although less extensively investigated, NLRC4 and NLRP12 appear to have an important role in the development of immune response against Leishmania in mice [6,15,16,17].

In humans, Moreira et al. [18] observed an increased AIM-2 expression in mucocutaneous lesions compared to localized lesions in ACL caused by *L. (V.) braziliensis*. These data suggest that the AIM2 inflammasome can be another mechanism in skin immune response and can affect the disease severity.

Given the complex immunopathogenesis of ACL and the current lack of studies showing the involvement of different inflammasome components across the broad clinical and immunopathological spectrum of ACL, our research aimed to conduct transcriptomic and immunopathological analyses to investigate the involvement of receptors previously indicated as key molecules in *Leishmania* response, AIM2, NLRC4, NLRP3, and NLRP12, together with main inflammasome components, namely caspase-1, caspase-5, GSDMD, IL-1β, and IL-18, in the different clinical forms of ACL caused by *L. (L.) amazonensis* and *L. (V.) braziliensis*.

## 2. Materials and Methods

### 2.1. Samples

We used 20 skin biopsies from patients with different clinical forms of ACL (5 ADCL caused by *L. (L.) amazonensis*, 4 LCL caused by *L. (L.) amazonensis*, 6 LCL caused by *L. (V.) braziliensis*, and 5 MCL caused by *L. (V.) braziliensis*). The patients were attended at the “Prof. Dr. Ralph Lainson” Leishmaniasis Laboratory of the Evandro Chagas Institute in Ananindeua, Pará State, Brazil, and treated after biopsy collection. Detailed patient characteristics, including sex, age, and disease duration are available in the Supplementary Data (S1). The patients were diagnosed by the Montenegro skin test, histopathological analysis, and molecular diagnosis. For the control group, we used six healthy skin biopsies (from a non-endemic area) obtained from plastic surgery remnants at the Medical Investigation Laboratory—Microsurgery and Plastic Surgery (LIM-04) HC/FMUSP. Written informed consent was obtained from individuals involved in the research, and this study was approved by the Ethics Committee of the Medicine School of São Paulo University, São Paulo State, Brazil (CAPPESQ-HC/FMUSP, CAAE: 03952418.3.0000.0065/Number: 3.080.387 and 3.950.475).

### 2.2. Histopathology

The skin fragments were obtained, processed, and stained with Hematoxylin-Eosin (HE). The prepared slides were examined under an Axioskop 2 Plus Zeiss optical microscope, focusing on a detailed histopathological assessment of the epidermis and dermis. The evaluation recorded the type and distribution of the inflammatory infiltrate, the predominant cell types, the presence or absence of granulomas, and any evidence of parasitism. A comparative and semi-quantitative histopathological analysis of the HE-stained sections was performed [19].

### 2.3. Immunohistochemistry

For immunohistochemistry, 4 µm-thick paraffin-embedded tissue sections mounted on pretreated slides were deparaffinized in xylene for 15 min and rehydrated in descending alcohol concentrations. Antigen retrieval was performed in a steam bath at 96 °C for 30 min, using a 10 mM citrate buffer (pH 6) for anti-NLRP3, AIM-2, NLRC4, NLRP12, caspase-1, caspase-5, GSDMD, and IL-1β antibodies, and 1 mM TRIS-EDTA buffer (pH 9) for the anti-IL-18 antibody. The sections were incubated with primary antibodies anti-NLRP3 (polyclonal, E-AB-70161, Elabscience, Houston, TX, USA, 1:600), anti-AIM-2 (polyclonal, E-AB-10974, Elabscience, 1:100), anti-NLRC4 (polyclonal, E-AB-67860, Elabscience, 1:100), anti-NLRP12 (polyclonal, E-AB-66298, Elabscince, 1:500), anti-caspase-1 (monoclonal, G6231-3D2, Sigma Aldrich, St. Louis, MO, USA, 1:500), anti-caspase-5 (monoclonal, MAA770Hu22, Cloud-clone Corp., Katy, TX, USA, 1:100), anti-GSDMD (monoclonal, E-AB-81473, Elabscience, 1:200), anti-IL-1β (polyclonal, ab2105, Abcam, Cambridge, MA, USA, 1:800), and anti-IL-18 (polyclonal, ab68435, Abcam, 1:2000). On the following day, the slides were incubated in a humid chamber at 37 °C with a post-primary antibody (NOVOLINK TM polymer detection systems—RE7280-K Leica Biosystems, Nussloch, Germany) and polymer from the same kit. Antigen–antibody binding was visualized by incubation with the DAB + H_2_O_2_ chromogenic substrate (diaminobenzidine with hydrogen peroxide—K3468—DakoCytomation). Finally, the slides were counterstained with Harris hematoxylin, dehydrated in ascending alcohol concentrations, and mounted with coverslips and synthetic resin.

### 2.4. Quantitative Analysis of Immunostained Cells

To determine the density of immunostained cells, 10 microscopic fields per slide were photographed using an Axioskop 2 Plus Zeiss microscope coupled to a computer with Axiovision 4.8 software (Zeiss, Oberkochen, Germany). Subsequently, the quantitative analysis of immunostained cells was performed. The mean number of immunostained cells per field was calculated, and then the cell density for each clinical case was determined based on the ratio between the mean number of immunostained cells in the 10 fields and the area of the microscopic field, which is 0.036852 mm^2^.

Descriptive statistics were performed, presenting means and dispersion. To assess statistical differences in cytokine expression among clinical forms of the ACL spectrum, one-way ANOVA was used. Shapiro–Wilk normality testing was conducted, and Dunn’s or Tukey’s post-hoc tests were applied depending on whether samples were non-parametric or parametric, respectively, adopting a significance level of *p* ≤ 0.05.

### 2.5. Transcriptome

For gene expression analysis, collected biopsies were preserved in RNAlater (Invitrogen, Waltham, MA, USA) and stored at −80 °C. Total RNA extraction from the tissue was carried out using the RNAeasy Mini kit (Qiagen, Valencia, CA, USA). Total RNA (500 ng) were used to prepare whole-transcriptome libraries using TruSeq Stranded Total RNA with Ribo-Zero Gold (Illumina, San Diego, CA, USA). Large-scale RNA sequencing was performed on the Illumina HiSeq 2000 platform. Following sequencing, the data underwent quality control using FASTQC 0.11.9 software [20], removing any data that did not meet pre-established quality standards. Next, reference-guided mapping and automatic gene annotation were performed to obtain functional information [21]. To understand the gene expression in different clinical and immunopathological groups, differential expression analysis of inflammasome-related genes was conducted.

## 3. Results

### 3.1. Histopathological Analysis

Histopathological analysis of samples from ADCL patients revealed an intense inflammatory infiltrate in the dermis, characterized by a predominance of vacuolated macrophages and high parasitic load. In LCL caused by *L. (L.) amazonensis*, a moderate/intense inflammatory infiltrate was also observed; however, it included macrophages, lymphocytes, and plasma cells, with moderate or mild parasitism and granuloma formation in some cases. In contrast, in LCL caused by *L. (V.) braziliensis*, the infiltrate was predominantly composed of lymphocytes and plasma cells, with few macrophages and mild parasitism. In MCL patients, an intense lymphoplasmacytic inflammatory infiltrate was noted, with scarce macrophages and few parasites. In some cases, well-formed granulomas were also observed. Epidermal changes were observed in 80% of the analyzed lesions and were mainly characterized by irregular acanthosis and frequent ulceration in MCL cases.

### 3.2. Transcriptome Analysis

**Figure 1:**
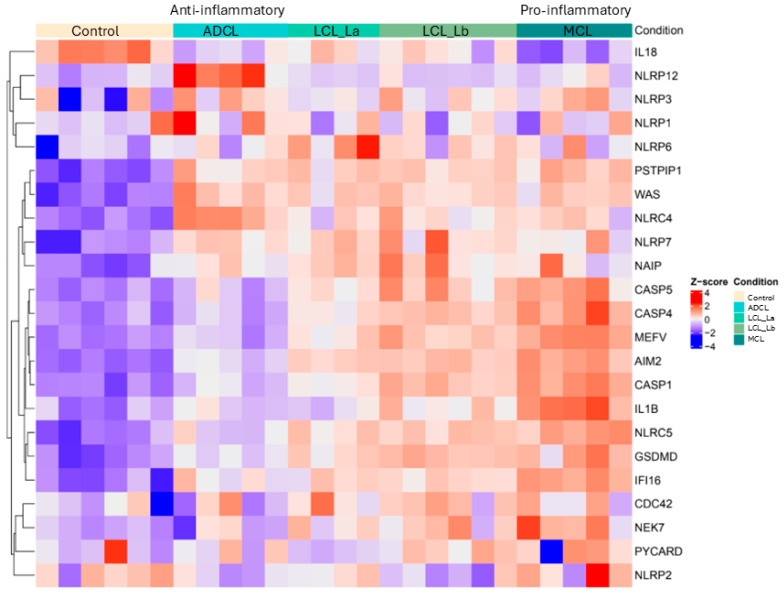
Heatmap showing transcription rates of inflammasome-related genes. The color scale represents the z-score for each gene. Higher gene expression relative to the mean corresponds to a higher z-score (red), while lower expression corresponds to a lower z-score (blue) in comparison to healthy individuals. ADCL (anti-inflammatory): Anergic Diffuse Cutaneous Leishmaniasis is caused by *L. (L.) amazonensis*; LCL (La): Localized Cutaneous Leishmaniasis is caused by *L. (L.) amazonensis*; LCL (Lb): Localized Cutaneous Leishmaniasis is caused by *L. (V.) braziliensis*; and MCL (pro-inflammatory): Mucocutaneous Leishmaniasis is caused by *L. (V.) braziliensis*.

Gene expression analysis revealed a positive regulation of important inflammasome-related genes (*NLRP3*, *AIM2*, *NLRP12*, *NLRC4*, *CASP1*, *CASP5*, *GSDMD*, and *IL1B)* in lesion samples of different clinical forms from ACL patients compared to the control group (Figure 1). *IL-18* gene was downregulated in the polar forms ADCL (anti-inflammatory) and MCL (pro-inflammatory). Although without immunohistochemical validation, other inflammasome-related genes such as *CASP4*, *MEFV*, *NLRC5*, and *IFI16* showed an upregulation in MCL, while in ADCL this regulation was downregulated.

**Figure 2:**
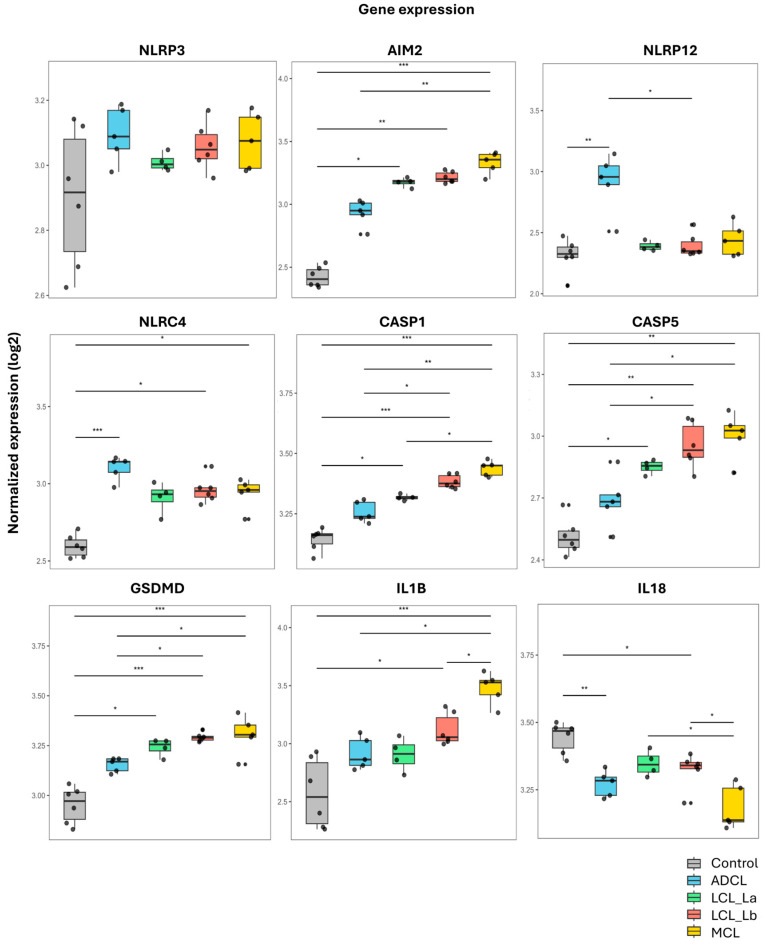
Boxplots showing normalized gene expression data from RNA-seq of lesions in patients with different clinical forms of American Cutaneous Leishmaniasis caused by *L. (L.) amazonensis* and *L. (V.) braziliensis.* ADCL: Anergic Diffuse Cutaneous Leishmaniasis, caused by *L. (L.) amazonensis*; LCL (La): Localized Cutaneous Leishmaniasis, caused by *L. (L.) amazonensis*; LCL (Lb): Localized Cutaneous Leishmaniasis, caused by *L. (V.) braziliensis*; and MCL: Mucocutaneous Leishmaniasis caused by *L. (V.) braziliensis.* A: NLRP3; B: AIM2; C: NLRP12; D: NLRC4; E: CASP1; F: CASP5; G: GSDMD; H: IL-18; I: IL-1 β. * = *p* < 0.05; ** = *p* < 0.001 and *** = *p* < 0.0001.

For better comparison of gene expression among the different clinical forms of ACL and the control group, boxplots were generated from the transcriptome analysis (Figure 2).

The data showed that the *NLRP12* inflammasome gene was higher expressed in ADCL compared to other clinical forms. In contrast, *AIM2*, *GSDMD*, *CASP1*, and *IL1B* genes were higher expressed in MCL. Interestingly, there was a negative regulation of IL-18 in patients compared to healthy controls.

**Figure 3:**
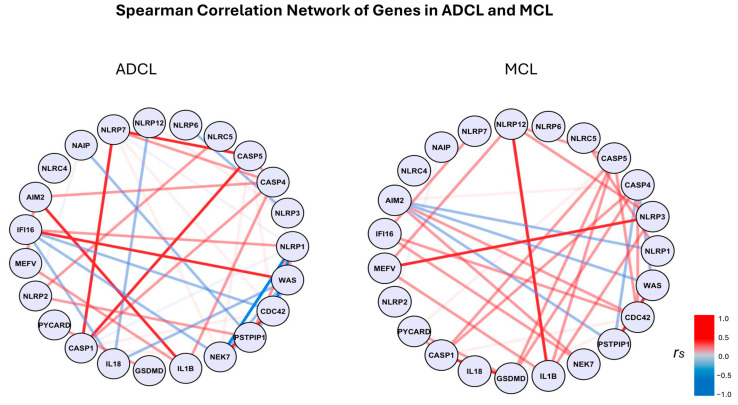
Spearman correlation networks of inflammasome-related genes in ADCL and MCL conditions. Each node represents a gene, and the edges indicate the correlation between them. The color of the edges represent the Spearman correlation coefficient (r_s_): red indicates positive correlation and blue indicates negative correlation. The intensity of the color corresponds to the magnitude of the correlation, with darker shades representing stronger correlation. The scale on the right shows the correlation range from −1 (strong negative correlation) to +1 (strong positive correlation).

Spearman’s correlation analysis (Figure 3) showed a strong positive correlation between *CASP1* and *CAPS5* (*p* < 0.05), and between *AIM2* and *IL1B* (*p* > 0.05) in ADCL. In MCL, there was a strong positive correlation between *NLRP12* and *IL1B* (*p* < 0.05), *CASP1* and *GSDMD* (*p* < 0.05), and *NLRP3* and *MEFV* (*p* < 0.05).

### 3.3. Immunohistochemistry Analysis

**Figure 4:**
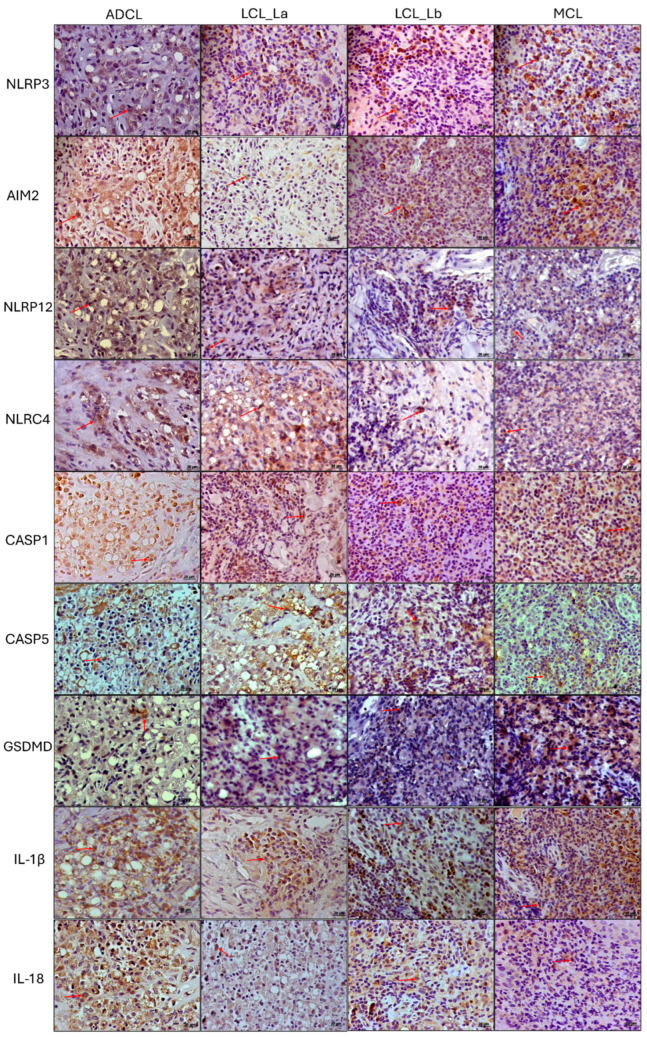
Immunohistochemistry reactions on histological sections of biopsies from patients with different clinical forms of ACL using anti-NLRP3, anti-AIM-2, anti-NLRP12, anti-NLRC4, anti-caspase-1, anti-caspase-5, anti-GSDMD, anti-IL-1β, and anti-IL-18 antibodies. ADCL: Anergic Diffuse Cutaneous Leishmaniasis caused by *L. (L.) amazonensis*; LCL (La): Localized Cutaneous Leishmaniasis caused by *L. (L.) amazonensis*; LCL (Lb): Localized Cutaneous Leishmaniasis caused by *L. (V.) braziliensis*; MCL: Mucocutaneous Leishmaniasis caused by *L. (V.) braziliensis*. Red arrows indicate immunostained cells.

**Figure 5:**
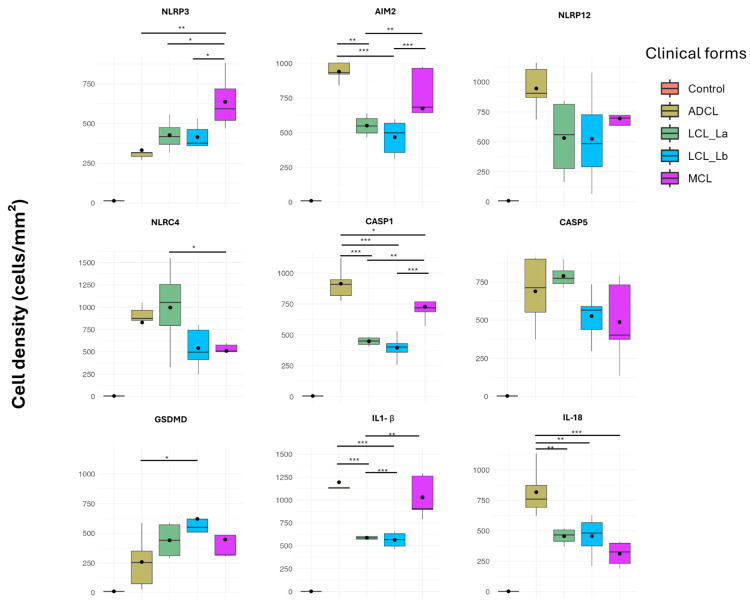
Boxplots showing the mean densities of cells (cells/mm^2^) expressing the various markers in histological sections of lesions from patients with different clinical forms of American Cutaneous Leishmaniasis caused by *L. (L.) amazonensis* and *L. (V.) braziliensis.* ADCL: Anergic Diffuse Cutaneous Leishmaniasis caused by *L. (L.) amazonensis*; LCL (La): Localized Cutaneous Leishmaniasis caused by *L. (L.) amazonensis*; LCL (Lb): Localized Cutaneous Leishmaniasis caused by *L. (V.) braziliensis*; MCL: Mucocutaneous Leishmaniasis caused by *L. (V.) braziliensis.* * = *p* < 0.05; ** = *p* < 0.001 and *** = *p* < 0.0001.

Immunopathological analysis of an ACL lesion was performed to validate the findings from the transcriptomic analyses. Photomicrographs of immunohistochemical reactions were examined, and graphs were generated showing the density of immunostained cells with the markers throughout the broad clinical and immunopathological spectrum of ACL (Figure 4 and Figure 5). Higher densities of AIM-2+, NLRP12+, CASP1+, and IL-1β+ cells were observed in both polar forms of the disease (ADCL and MCL) compared to the localized forms (Figure 5). There was higher in situ expression of NLRP3 in the hyperreactive form (MCL) in comparison to the other clinical groups, whereas a higher density of IL-18+ cells was found in the hyporeactive form (ADCL) compared to the other forms. NLRC4+ cells were found at higher densities in both clinical forms caused by *L. (L.) amazonensis* (ADCL and LCL La). Evaluation of in situ GSDMD expression showed lower densities of immunostained cells in ADCL compared to other forms in the ACL spectrum. The cell density values of immunostained cells with nine inflammasome markers are shown in Table 1. The epidermis displayed immunostaining for AIM2 and NLRP12 receptors, with greater intensity in the basal layer, especially in the polar forms (ADCL and MCL). The staining intensity varied from case to case and decreased alongside keratinocyte maturation.

## 4. Discussion

*L. (L.) amazonensis* and *L. (V.) braziliensis* are responsible for a broad spectrum of clinical and immunopathological manifestations in human cutaneous leishmaniasis [3], ranging from the central form (LCL) to the more severe extreme forms, ADCL and MCL. According to Silveira et al. [4,5], this clinical-immunopathological spectrum is strongly associated with the *Leishmania* species, its virulence, and the host’s immunogenetic profile. A recent study [22] demonstrated the role of important genes related to the inflammatory process in the spectrum of ACL; however, the involvement of inflammasome genes in cutaneous leishmaniasis has not yet been sufficiently investigated, representing a promising area for research [23]. Given this gap of studies covering the entire clinical-immunopathological spectrum of the disease caused by *L. (L.) amazonensis* and *L. (V.) braziliensis* and considering the complexity of the immunopathogenesis of ACL, this study aimed to analyze the participation of inflammasomes through transcriptomic and immunopathological evaluations, building upon our previous research and contributing to a better understanding of the disease.

As expected, skin leishmaniasis lesions exhibit increased expression of inflammasome components compared to normal skin, with the highest levels observed in the hyperreactive form of the lesion (MCL). Skin cells display different expression profiles of inflammasome components, with AIM2 and NLRP12 expected to be highly expressed by keratinocytes, while NLRP3 and NLRC4 are predominantly expressed in innate immune cells. Recent literature has also demonstrated the role of AIM2, NLRP12, and NLRP3 in lymphocytes [24,25,26].

Therefore, the increased expression levels of inflammasome components in skin lesions may result from the response of tissue cells (keratinocytes and resident immune cells) to infection, as well as from the infiltration of circulating leukocytes. In inflammatory skin diseases such as psoriasis and atopic dermatitis, AIM2 and NLRC4 are overexpressed in skin lesions, suggesting a common inflammasome expression profile during skin inflammation [27,28,29]. Intriguingly, in these diseases, GSDMD is also found to be upregulated in skin lesions, similar to what we observed in most inflammatory ACL lesions [30,31].

Activation of the NLRP3 inflammasome is a key mechanism in the pathogenesis of several inflammatory diseases and is currently considered a potential therapeutic target [32]. In our study, transcriptomic analysis revealed increased NLRP3 gene expression in the hyperreactive clinical form (MCL) compared to control, ADCL, and LCL forms, as well as a higher density of immunostained cells (NLRP3+) in situ when compared with other ACL forms. Activation of the NLRP3 inflammasome and IL-1β production are associated with cytotoxicity and tissue damage in patients with LCL caused by *L. (V.) braziliensis* [33]. A similar phenomenon may be occurring in MCL, also caused by the same species, since we detected high *IL1B* expression and a greater density of IL-1β+ cells in the tissue. Therefore, NLRP3 and IL-1β+ may contribute to the exacerbation of the inflammatory process in these patients.

The AIM2 inflammasome is a cytoplasmic sensor that recognizes the genetic material of invading pathogens. Once activated, it contributes to physiological responses against infection; however, its dysregulation can favor the progression of autoimmune and autoinflammatory diseases [34]. In ACL, our data revealed higher *AIM2* gene expression in MCL and higher protein densities in both of the polar and more severe forms (ADCL and MCL) compared to localized forms. In clinical forms caused by *L. (V.) braziliensis*, AIM2 was associated with disease severity in a study which compared gene expression in localized and mucocutaneous lesions [18], corroborating our gene and protein analyses in the *L. (Viannia)* subgenus. Regarding the *L. (Leishmania)* subgenus, this is the first study to show AIM2 involvement in ADCL caused by *L. (L.) amazonensis*. Considering that high levels of AIM2+ cells were found in situ, we suggest that AIM2 activation, together with Th17, Treg, and Th2 cells [3,35] may contribute to suppressing the cellular immune response in these patients, although further studies are needed to explore this inference.

Cai et al. [36] demonstrated a link between NLRP12 and the production of the pro-inflammatory cytokine IL-17, contributing to neutrophil recruitment and bacterial infection control in mucosal tissue. However, other studies indicate that NLRP12 may play an anti-inflammatory role, suggesting a dual function [17]. ADCL results from a hypo-response to the pathogen, characterized by a Th2 immune response. The increased NLRP12 expression observed in our ADCL samples aligns with recent literature highlighting the role of NLRP12 in regulating the NLRP3 inflammasome in keratinocytes [37,38]. Moreover, an intrinsic role of NLRP12 in shaping T cell differentiation has been demonstrated. NLRP12 deficiency promotes a hyperinflammatory T cell response [39], suggesting that when NLRP12 is upregulated, it may negatively regulate the Th1/Th17 response in favor of a Th2 response, which is expected in ADCL. In the hyperreactive form (MCL), immunohistochemistry also revealed a high density of NLRP12+ cells, reinforcing the hypothesis that this inflammasome may play both pro- and anti-inflammatory roles depending on the biological context (antigenic microenvironment: *L. (V.) braziliensis* or *L. (L.) amazonensis*, respectively).

As for the NLRC4 inflammasome, we found higher gene transcription in the ADCL and higher protein expression in situ in both clinical forms caused by *L. (L.) amazonensis* (ADCL and LCL). Recently, the NLRC4 inflammasome was associated with host susceptibility in infectious diseases, mainly due to its ability to inhibit nitric oxide secretion within macrophages, leading to poor parasite control [16]. Furthermore, the same study showed that NLRP3 may be inhibited by NLRC4. These findings corroborate our current results, where we found a higher density of NLRC4+ cells in both clinical forms (ADCL and LCL) characterized by the presence of vacuolated, parasite-rich macrophages due to the inactivation of their leishmanicidal mechanisms. Considering the aforementioned studies, our data also suggests that NLRP3 may be inhibited by NLRC4 in these two clinical forms, as they presented lower densities of NLRP3+ cells and higher densities of NLRC4+ cells.

Inflammasome sensors detect a stress signal, and inflammatory caspases are essential for activating the inflammasome complex. Among the different types of caspases, caspase-1 and caspase-5 are related to canonical and non-canonical activation, respectively. Here, the transcriptome analysis showed that both genes coding caspase-1 and caspase-5 displayed progressively increased expression across the disease spectrum, starting from the hyporeactive form (lowest expression) to the hyperreactive form (highest expression). Once inflammasomes are activated by caspases, GSDMD is cleaved, forming pores in the plasma membrane that allow the secretion of IL-1β and IL-18 into the extracellular environment. Notably, inflammasome-dependent cytokines IL-1β and IL-18 show opposite expression levels in the extreme response profiles of ADCL and MCL. In line with the expected homeostatic role of IL-18 in the skin, ADCL lesions exhibit the highest levels of this cytokine, whereas in the hyperreactive MCL form, IL-1ß predominates. A strong positive correlation was observed between the genes AIM2 and IL1B (ADCL) and between NLRP12 and IL1B (MCL), suggesting that cytokine expression is increased when these inflammasomes are activated. Moreover, a strong positive correlation was observed between CASP1 and CASP5 in ADCL, and between CASP1 and GSDMD in MCL, indicating that in the hyporeactive clinical form (ADCL), inflammasomes are activated by both canonical and non-canonical pathways, whereas in MCL, the main activation occurs via the canonical pathway.

IL-1β was associated with disease severity in a study with *L. (L.) mexicana*, as higher serum levels of this cytokine were found in patients with ADCL in comparison to the patients with LCL [40]. Interestingly, other researchers also linked IL-18 to disease severity caused by *L. (L.) amazonensis*, as IL-18-deficient mice developed smaller lesions and lower parasite loads compared to wild-type animals, suggesting this cytokine’s contribution to host susceptibility to infection [10]. In our study, we detected high densities in situ of IL-1β+ and IL-18+ cells in the polar ADCL form caused by the *L. (Leishmania)* subgenus (*L. (L.) amazonensis*). These data align with the aforementioned findings [10,14,40], as this clinical form is characterized by host susceptibility to infection and a predominant Th2-type cellular immune response.

On the other hand, high IL-1β+ densities were found in skin fragments from patients with MCL caused by *L. (V.) braziliensis*, further highlighting the role of the inflammasome in disease severity [18]. In addition, in a previous study conducted in our laboratory, Gonzalez et al. [41] analyzed inflammasome involvement in LCL caused by *L. (V.) panamensis* and found caspase-1 and IL-1β at the lesion site, suggesting that inflammasome activation is crucial for controlling parasitism, given the inverse correlation between IL-1β and the number of amastigotes. These data reinforce the role of IL-1β in parasite control while not excluding its contribution to disease severity. Our findings corroborate the above-mentioned studies as we observed a rare parasitism in HE sections and high IL-1β expression in the hyperreactive clinical form caused by *L. (V.) braziliensis.*

Recently, de Sá et al. [12] showed that GSDMD is important for parasite restriction in experimental infections caused by different *Leishmania* species. However, an interesting finding revealed that in *L. (L.) amazonensis* infection, weak GSDMD activation can lead to temporary pore formation in the plasma membrane without causing macrophage death by pyroptosis. Thus, the authors hypothesized that this could be another parasite escape mechanism, contributing to persistent infection. In our work, both immunohistochemistry and transcriptomic analysis showed lower GSDMD expression in ADCL caused by *L. (L.) amazonensis* in comparison to the other forms. Considering that the histopathological analysis showed a macrophage-rich inflammatory infiltrate heavily parasitized, and given that this clinical form is characterized by an anergic immune response, the persistence of the infection may also be linked to the parasite escape mechanism mentioned above.

Overall, this study demonstrated that the activation of the NLRP3, AIM-2, NLRP12, and NLRC4 inflammasomes is distinctly associated with different clinical forms of ACL caused by *L. (L.) amazonensis* and *L. (V.) braziliensis*. In particular, the high densities of NLRP3+, AIM-2+, and IL-1β+ cells found in the hyperreactive form of the disease suggest a role in exacerbating the inflammatory response in patients with mucocutaneous leishmaniasis (MCL). Conversely, in more severe forms like ADCL, increased expression of AIM-2, NLRP12, and NLRC4, combined with reduced GSDMD activation, may indicate an immune-evasion mechanism contributing to parasite persistence. These findings enhance our understanding of the role of inflammasomes in the clinical-immunopathological spectrum of ACL. Nevertheless, further research is required to develop and evaluate therapeutic strategies capable of selectively targeting inflammasomes.

In ADCL, such strategies should aim to restore effective immune responses, potentially by reactivating inflammasome pathways through the activation of GSDMD, for example. In contrast, in MCL, inhibition of NLRP3 inflammasome activation or blockade of IL-1β signaling with agents such as Anakinra or Canakinumab could attenuate the exaggerated inflammatory response [42,43,44]. It is important to emphasize that these treatments are already available and have been proposed for use in inflammatory skin diseases [45]. However, given the ubiquitous expression and critical immunological functions of inflammasomes, systemic modulation carries the risk of broad immunosuppression and off-target effects. Therefore, the development of localized, lesion-targeted therapies—such as topical formulations or intralesional delivery—represents a safer and more precise therapeutic approach. This perspective opens new avenues for future studies aimed at designing immunomodulatory strategies that are both effective and context-specific for the treatment of cutaneous leishmaniasis.

## Figures and Tables

**Table 1:** Mean densities (mean ± standard error) of NLRP3+, AIM-2+, NLRC4+, NLRP12+, CASP1+, CASP5+, GSDMD+, IL-1β+, and IL-18+ cells in the different clinical forms of ACL caused by *L. (L.) amazonensis* (La) and *L. (V.) braziliensis* (Lb) and in the health control.

Cell Density (Cells/mm^2^ ± Standard Error)					
Antibody (Marker)	Control	ADCL	LCL (La)	LCL (Lb)	MCL
NLRP3	13.56 ± 3.32	332.04 ± 29.90	427.13 ± 44.03	414.95 ± 28.22	636.91 ± 55.30
AIM2	8.47 ± 3.10	941.21 ± 27.05	551.05 ± 33.93	467.56 ± 47.55	869.97 ± 64.49
NLRC4	5.27 ± 1.11	827.94 ± 108.18	995.73 ± 221.50	541.94 ± 83.74	509.61 ± 30.17
NLRP12	8.29 ± 2.20	945.57 ± 76.09	531.88 ± 148.16	525.15 ± 137.03	695.60 ± 86.49
CASP1	6.03 ± 2.73	914.23 ± 53.76	448.16 ± 15.73	396.48 ± 33.85	728.57 ± 40.20
CASP5	3.01 ± 3.49	689.12 ± 91.74	789.98 ± 35.11	526.65 ± 57.55	487.35 ± 108.99
GSDMD	10.61 ± 2.65	258.10 ± 90.10	440.73 ± 68.97	620.52 ± 108.68	447.27 ± 87.23
IL1-β	2.26 ± 1.11	1194.13 ± 71.32	587.19 ± 7.61	563.49 ± 31.39	1028.96 ± 76.73
IL-18	5.27 ± 2.12	816.65 ± 80.10	455.57 ± 30.08	456.13 ± 58.10	310.93 ± 32.82

## Data Availability

Data supporting the findings of the present study are available from the corresponding author upon request.

## Supplementary Materials

The following supporting information can be downloaded at: https://www.mdpi.com/article/10.3390/microorganisms13050980/s1, Table S1: Clinical and demographic data.
