# Supplementary material for: Transcriptomic and Immunopathological Profiles of Inflammasomes in Different Clinical Forms of American Cutaneous Leishmaniasis
Source: Microorganisms. 2025 Apr 24;13(5):980. doi: 10.3390/microorganisms13050980 (PMC12114145; doi:10.3390/microorganisms13050980)
Supplement: Supplementary file 1 [file microorganisms-13-00980-s001.zip › microorganisms-3543300-supplementary.pdf]

Clinical form (n)	Histopathological Characteristics			
	**Inflammatory infiltrate**			
	**Mild n(%)**	**Moderate n(%)**	**Intense n(%)**	
**ADCL [La] (n=5)**	1 (20%)	0 (0%)	4 (80%)	
**LCL [La] (n=4)**	0 (0%)	1 (25%)	3 (75%)	
**LCL [Lb] (n=6)**	0 (0%)	2 (33.3%)	4 (66.7%)	
**MCL [Lb] (n=5)**	0 (0%)	0 (0%)	5 (100%)	
	**Infiltrate Distribution**			
	**Focal n(%)**	**Diffuse n(%)**		
**ADCL [La] (n=5)**	1 (20%)	4 (80%)		
**LCL [La] (n=4)**	1 (25%)	3 (75%)		
**LCL [Lb] (n=6)**	3 (50%)	3 (50%)		
**MCL [Lb] (n=5)**	0 (0%)	5 (100%)		
	**Granuloma**			
	**Absent n(%)**	**Outline n(%)**	**Well-formed n(%)**	
**ADCL [La] (n=5)**	4 (80%)	1 (20%)	0 (0%)	
**LCL [La] (n=4)**	2 (50%)	0 (0%)	2 (50%)	
**LCL [Lb] (n=6)**	3 (50%)	2 (33.3%)	1 (16.67%)	
**MCL [Lb] (n=5)**	2 (40%)	1 (20%)	2 (40%)	
	**Parasitism**			
	**Negative n(%)**	**Mild n(%)**	**Moderate n(%)**	**Intense n(%)**
**ADCL [La] (n=5)**	0 (0%)	0 (0%)	0 (0%)	5 (100%)
**LCL [La] (n=4)**	0 (0%)	2 (50%)	2 (50%)	0 (0%)
**LCL [Lb] (n=6)**	3 (50%)	3 (50%)	0 (0%)	0 (0%)
**MCL [Lb] (n=5)**	3 (60%)	2 (40%)	0 (0%)	0 (0%)
